# Effectiveness of interventions for the prevention or management of workplace violence in student nurses during clinical placement: A systematic review

**DOI:** 10.1111/jan.16357

**Published:** 2024-07-30

**Authors:** Hila Ariela Dafny, Nasreena Waheed, C. J. Cabilan, Sandra Johnston, Vincent Pearson, Anne Mette Adams, Craig Phillips, Shannon Brown, Christine McCloud

**Affiliations:** ^1^ College of Nursing and Health Sciences Flinders University Bedford Park South Australia Australia; ^2^ Caring Futures Institute Flinders University Bedford Park South Australia Australia; ^3^ Mparntwe Centre for Evidence in Health Flinders University: A JBI Centre of Excellence Alice Springs Northern Territory Australia; ^4^ College of Nursing Charles Darwin University Darwin Northern Territory Australia; ^5^ Work Health and Safety Occupational Violence Prevention and Management, Work Health and Safety, Canberra Health Services Canberra Australian Capital Territory Australia; ^6^ School of Nursing, Midwifery and Social Work University of Queensland Brisbane Queensland Australia; ^7^ School of Nursing Queensland University of Technology Brisbane Queensland Australia; ^8^ JBI, School of Public Health, Faculty of Health and Medical Sciences The University of Adelaide Adelaide South Australia Australia; ^9^ Research Engagement Team Flinders University, Library Adelaide South Australia Australia

**Keywords:** bullying, clinical placements, management, nursing, nursing students, systematic review, workforce issues, workplace violence

## Abstract

**Aim:**

To systematically investigate the effectiveness of interventions for managing workplace violence experienced by registered nursing students during clinical placement.

**Design:**

A systematic review of experimental studies.

**Methods:**

The review was conducted according to the Preferred Reporting Items for Systematic Reviews and Meta‐Analyses guidelines. The key search concepts such as “Nursing students”, “Education”, “workplace violence”, “clinical placement” and “clinical study” were inspected to identify relevant articles (Appendix A). Two independent reviewers completed screening, critical appraisal and data extraction. Due to heterogeneity among the included studies, results were synthesized narratively.

**Data Sources:**

MEDLINE (Ovid), CINAHL (EBSCOhost), Web of Science Core Collection (Clarivate Analytics), Scopus (Elsevier), Embase (Ovid), Cochrane CENTRAL, ERIC (ProQuest), ProQuest Central and ProQuest Social Science Premium Collection were searched from inception to 27th February 2023.

**Results:**

A total of 13 studies were included in this review. The predominant intervention for managing workplace violence experienced by registered nursing students during clinical placements was education. Approaches varied among studies and included didactic teaching, e‐learning, role‐playing and simulation practice. The included studies showed uncertain improvements in registered nursing students' confidence, coping skills, knowledge, competence and self‐efficacy in dealing with workplace violence during clinical placements. Only one study assessed the incidence rate of workplace violence and found that a multi‐faceted intervention involving both staff and students decreased the incidence.

**Conclusion:**

Given the heterogeneity of educational interventions, the effect of interventions for managing workplace violence during students' clinical placement is uncertain. To address this gap, high‐quality, proactive and combined interventions at both institutional and organizational levels are needed.

## 1 INTRODUCTION

Violence in the workplace has become a worldwide problem, with healthcare professionals, especially nurses, facing increasing incidents in the clinical context (Cebrino & Portero de la Cruz, 2020). Workplace violence (WPV) is defined as “incidents where staff are abused, threatened, or assaulted in circumstances related to their work, including commuting to and from work, involving an explicit or implicit challenge to their safety, well‐being, or health” (International Labour Organization, International Council of Nurses, World Health Organization, International Labour Organization, International Council of Nurses, World Health Organisation, Public Services International, 2002). Nurses frequently report experiencing WPV, which includes a spectrum of incidents from physical and non‐physical violence to bullying and sexual harassment (Dafny & Beccaria, 2020). Workplace violence contributes to significant personal stress, discouragement and low self‐esteem (Dafny et al., 2022), often causing individuals to question their career choices (Kim et al., 2018).

The pervasive impact of WPV extends to all levels of nursing staff, including registered nursing students (RNS) who are completing their education programmes. As part of their education programmes, RNS are required to complete a requisite number of clinical placement hours (Pullen & Ahchay, 2022). These placements offer experiences in the clinical setting, which are essential for learning practical skills, applying knowledge and understanding professional behaviours within a healthcare environment (Simpson & Sawatzky, 2020). Similarly, RNS experience bullying, racism, discrimination and verbal and physical violence, and it impacts RNS widely, including psychological trauma, feeling hopeless, stressed and anxiety, reducing their academic performance and questioning their future in the nursing profession (Dafny et al., 2023; Hallett et al., 2023). RNS have specific vulnerabilities predisposing them to WPV during clinical placement, including being underprepared for placements concerning WPV in particular. Most of them are young; 72% of RNS are 20–39 years old, and with limited skills needed to identify and de‐escalate WPV. They are also unsupported by university facilitators (Johnston et al., 2024). RNS represent the future workforce, and it is crucial to identify effective interventions to prevent WPV during clinical placement and ensure a safe clinical learning environment. This review aims to contribute new knowledge by investigating effective interventions to address workplace violence towards RNS. The goal is to create a safer workplace environment for RNS and nurses, which could improve their retention and attraction to the nursing profession and help address the anticipated nursing workforce crisis.

## 2 BACKGROUND

Nurses frequently face WPV, with 44.6% of nurses experiencing at least one episode a year (Cheung & Yip, 2017). The prevalence and frequency of WPV against healthcare workers vary based on location, as indicated in the systematic review of Liu et al. (2019). The highest prevalence is in Australasia, followed by North America, Asia and Africa, and the lowest is in Europe (Liu et al., 2019). However, the true prevalence of WPV towards nurses is much higher, as many incidents go underreported. Babiarczyk et al. (2020) revealed that 70% of WPV incidents against nurses go unreported, as victims often believe reporting to be futile and unlikely to lead to any action. Registered nursing students face additional barriers in reporting WPV, fearing academic repercussions (Birks et al., 2018) and possible offender retaliation (Üzar‐Özçetin et al., 2021). Studies have identified a range of WPV perpetrators against RNS, including patients, visitors and fellow nurses (Authement, 2016; Cheung & Yip, 2017). It was also found that 60% to 100% of RNS experienced WPV directly or witnessed it against another student (Üzar‐Özçetin et al., 2020).

The detrimental impact of WPV on RNS is evident in several key areas, including a noticeable drop in academic performance compounded by ongoing stress and anxiety, along with a significant decrease in confidence regarding their future in the nursing profession (Dafny et al., 2023). Registered nursing students who were victims of WPV reported that such events notably compromised their ability to learn and perform in clinical settings (Thomas et al., 2015). This impact was further elaborated by Minton and Birks (2019), who pointed out that RNS experienced heightened levels of anxiety, not only during their clinical placements but also on completion. The cumulative effect of these experiences was profoundly damaging, as the trauma from viewing or experiencing WPV was often so severe that it led many RNS to question their decision to pursue a career in nursing, with some opting to leave the profession altogether (Üzar‐Özçetin et al., 2021). This worrying trend highlights the urgent need to address WPV in healthcare settings, to protect the well‐being of RNS and ensure the sustainability of the nursing profession.

The prevention and management of WPV can be achieved by enhancing awareness and improving communication and de‐escalation skills (Dafny & Muller, 2022). The efficacy of various strategies to prevent or manage WPV among healthcare workers has been the subject of several reviews (Spelten et al., 2020). Yet, there is no clear consensus on the most effective approach, and the impact of these strategies on RNS has received limited attention. Solorzano Martinez and De Oliveira (2021) mixed methods review revealed that training programmes focused on preventing and managing WPV were positively received by RNS and effectively enhanced their ability to handle violent scenarios. However, the review did not address the more subtle forms of violence, such as bullying and incivility. The proposed review aims to deepen current understanding by exploring practical ways to prevent WPV towards RNS during clinical placement, broadening its scope to encompass all forms of WPV and a comprehensive range of prevention and management strategies. The objective is to identify the most effective WPV prevention and management strategies and the support required for RNS following incidences of WPV.

## 3 THE REVIEW

### 3.1 Aim

The aim of this review was to systematically investigate the effectiveness of workplace violence management or prevention interventions, including strategies, protocols and policies, to address violence against and provide support to registered nursing students (RNSs) following incidents of violence during clinical placement.

As indicated in the a priori published protocol (Dafny et al., 2024), this systematic review aimed to define two outcome measures used to determine whether a programme was effective. The primary outcome was the number of WVP incidents towards RNSs during their clinical placement, resulting in (i) no harm or injury, (ii) psychological harm or injury or (iii) physical harm or injury. The primary outcome was measured using the Perception of Aggression Scale or lower incidence of the WPV report.

The secondary outcomes were to include levels of preparedness in recognizing and addressing any WPV, confidence, attitude and skills in managing incidents of WPV, as well as knowledge of the ability to cope with WPV. The secondary outcomes include knowledge, confidence, self‐efficacy, competence and attitude. The preparedness to manage WPV, was measured using the Management of Aggression and Violence Attitude Scale, the Confidence in Coping with Patient Aggression Instrument, the De‐escalating Aggressive Behaviour Scale, or investigator‐developed questionnaires.

The two outcomes, namely the “patient‐on‐student” group and the “staff‐on‐student” group, were identified based on the perpetrator focus of each study. The timing of the outcome assessment was variable, categorized as immediate, short‐term (within 3 months), or long‐term (≥3 months) from intervention completion. This categorization provides a clear structure for understanding the research design and the progression of the study.

### 3.2 Design

This review was conducted in accordance with the JBI's Systematic Review of Effectiveness method, an a priori published protocol (Dafny et al., 2024) and a registered PROSPERO protocol (CRD42022377318). This review was reported according to the Preferred Reporting Items for Systematic Reviews and Meta‐Analyses (PRISMA) guidelines (Page et al., 2021).

### 3.3 Search methods

An initial limited search of MEDLINE (via Ovid) and CINAHL (via EBSCO) was performed to identify keywords and index terms, which was then used to develop a complete search strategy using appropriate search terms and Medical Subject Headings (MeSH) demonstrated in the search strategy (Appendix A).

**FIGURE 1:**
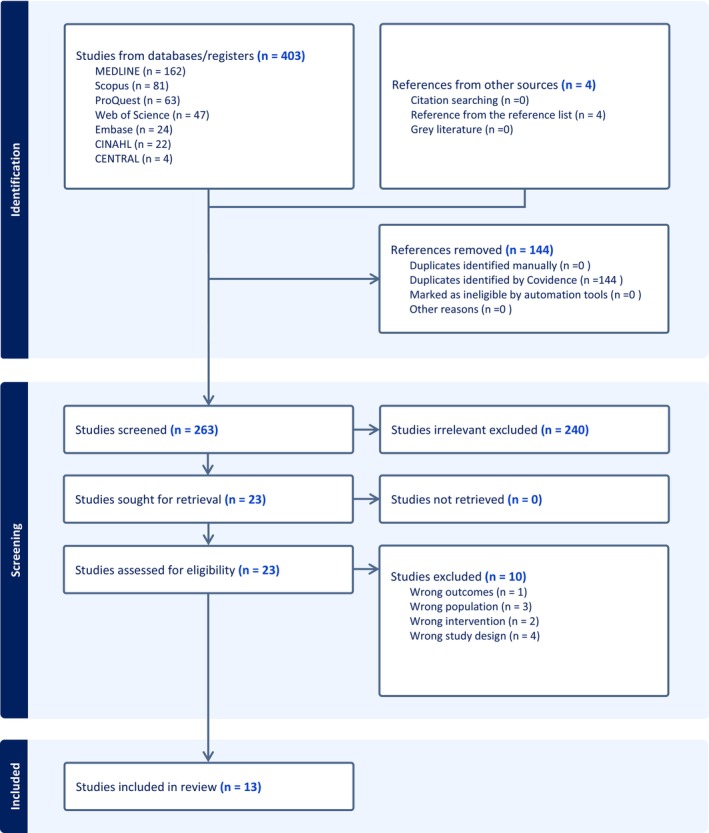
PRISMA flow diagram of the literature search and selection procedure for inclusion studies.

The final search strategy, informed by the preliminary search results and keywords developed, ensured the identification of all relevant articles from inception to 27 February 2023. All keywords and index terms were adapted for the bibliographic databases MEDLINE (Ovid), CINAHL (EBSCOhost), Web of Science Core Collection (Clarivate Analytics), Scopus (Elsevier), Embase (Ovid), Cochrane CENTRAL, ERIC (ProQuest), ProQuest Central and ProQuest Social Science Premium Collection and run on 27 February 2023. A supplemental search was conducted on Google Scholar. Sources of unpublished studies and grey literature were searched, including ClinicalTrials.gov, ProQuest Dissertations and Theses Global, TROVE, the Networked Digital Library of Theses and Dissertations (NDLTD), the World Health Organization International Clinical Trial Registry Platform and the National Institute for Health and Care Excellence (see Figure 1 Identification section of PRISMA flow chart). The reference lists of all the studies selected for screening were examined for additional studies. Two hundred and sixty‐three studies progressed to the screening phase of the search (see Figure 1.PRISMA flow chart—Identification).

### 3.4 Inclusion and exclusion criteria

To capture all relevant articles, this review included published and unpublished studies in any language assessing the effectiveness of policies, protocols, interventions, or strategies aiming to address WPV towards RNSs. The following inclusion and exclusion criteria were developed.

#### 3.4.1 Inclusion criteria

- A focus on RNS undertaking a formal education to become a registered nurse. There were no limitations in terms of gender, age, length of time spent on placement or type of education programme. (b) Studies reporting on educational interventions and pre‐clinical placement strategies measuring the preparedness and confidence of RNSs.
- Quantitative experimental studies measuring the effectiveness of implemented policies, protocols, interventions, or strategies such as randomized controlled trials (RCTs), controlled before and after, interrupted time series and pre‐test‐post‐test studies.
- The primary outcome of interest was the number of workplace violence incidents towards RNS during their clinical placement, resulting in (1) no harm or injury, (2) psychological harm or injury or (3) physical harm or injury. The secondary outcomes were levels of preparedness in recognizing and addressing any WPV, and level of attitude, knowledge and confidence related to recognizing, addressing and coping with WPV (Dafny et al., 2024).

#### 3.4.2 Exclusion criteria

- Papers that were not focused on RNS in the clinical setting.
- Not experimental in design.
- Not concerned with WPV prevention or management.

### 3.5 Search outcomes

A total of 403 articles were identified, leaving 144 after removing duplicates (Figure 1 PRISMA flow chart). These articles were imported to Covidence (Veritas Health Innovation, Melbourne, Australia), and the titles were screened for relevancy to the review. Abstracts were then reviewed by two reviewers independently against the inclusion and exclusion criteria for progression in the search. When a determination was not made based on the abstract, the full paper was reviewed to make a judgement. Any disagreements between the reviewers at each stage of the study selection process were resolved through discussion. Reference lists of each retrieved paper were examined to identify any further studies. A total of 13 papers progressed to critical appraisal before final inclusion in the review.

### 3.6 Quality appraisal

Studies meeting the inclusion criteria were appraised by two independent reviewers using the JBI critical appraisal tools for randomized controlled trials or quasi‐experimental studies (Tufanaru et al., 2020). Furthermore, the revised JBI critical appraisal tool (Barker et al., 2024) was also used to accurately summate the risk of bias and internal validity of the reviewed papers. After appraisal, discrepancies between the two reviewers were resolved through discussion. The pairs discussed the appraisal results to reach a consensus. If no agreement was achieved, the third reviewer resolved the discrepancies.

All 13 papers were included irrespective of their methodological quality. In adherence with the published protocol (Dafny et al., 2024), data extraction was conducted for all papers, irrespective of their quality (see Table 4). The use of all papers, regardless of quality, provides a true reflection of the current literature on a specific topic (Barker et al., 2024).

### 3.7 Data extraction

Initially, data relating to interventions to prevent workplace violence and the outcomes were extracted by two reviewers independently and then compared to ensure accuracy. Data were then extracted into summary tables created using JBI SUMARI (Munn et al., 2018) and Covidence (Veritas Health Innovation, Melbourne, Australia) as a guide. Further data extraction was conducted by teams of two reviewers allocated to specific studies. For each study, data were tabulated using the following variables: Characteristics of included studies (Table 1) including the first author, year of publication and papers' title, country of context and target population, methodology and type of intervention, theoretical constructs, measurement scales, data collection methods and analysis methods (Table 1). The Summary of Findings Table 4 presented the outcomes based on the perpetrator focus of each study: the “patient‐on‐student” group and the “staff‐on‐student” group and focused on the intervention of each included study, including the content and mode (face‐to‐face, simulation, lecturers, role play, etc.), the timing of the immediate, short‐term outcomes (up to 3 months post‐intervention as most of the studies durations were short‐term) and the summary of the included studies' findings (Table 4). Table 5 displayed the detailed summary of the findings and included further details regarding the interventions (such as number of participants, delivery schedule, time between interventions and time of last measurement) and core constructs that were represented in each reviewed paper (knowledge, confidence, self‐efficacy, competence and attitude) and the level of significance (Table 5).

**TABLE 1:** Characteristics of included studies.

Papers	Country of context and target population	Methodology and type of intervention	Duration of intervention and study	Theoretical constructs used	Measurement scales used	Data collection methods	Analysis methods
Beech & Leather, 2003	The United Kingdom 243 nursing students	Pre‐post‐test design; Repeated measures longitudinal design	3‐day education unit consists of knowledge of psychological theories and models, attitudes, increasing self‐esteem, and breakaway skills. Duration of intervention: 3 days. Follow‐up: 8 months	Kraiger et al. (1993) Cognitive, skill based, and affective Theories of learning outcomes	A purpose designed questionnaire to measure changes in specified domains of learning outcomes,	Questionnaire administered at four time points. The questionnaire captures demographic data and participants' responses to two aggression scenarios. The questions requested that the respondent identify risk factors within the scenario and used open responses to measure declarative knowledge and evidence of more expert mental mapping. Next, a 10 cm line VAS was used to measure attribution of blame to the patient in each case. 24 likert‐typle statements were included to measure attitudes, behavioural competence, confidence, belief in myths.	Exploratory factor analysis to analyse statements. Principal component analysis to exam themes within the statements. Matched pair *t*‐test.
Cherian & Sharma, 2019	India 71 nursing students	Pre‐test‐post test A training programme on aggression management. No info on the components of this programme	Not stated	Not stated.	self‐administered questionnaire and Likert scale to assess the level of knowledge and confidence	Data were collected in two phases, Phase 1 collected baseline data. Phase 11 collected prior to the intervention and 1 week after the intervention	Statistical analysis—paired *t*‐test for comparison and Chi square test
Jeong & Lee, 2020	South Korea Total of 45 nursing students 22 nursing students assigned to the experimental group 23 nursing students assigned to the control group	Pre‐post (non‐equivalent)	8 sessions, 120 min per session, two sessions per week for 4 weeks. 3 months follow‐up	Kim (2002) Conceptual model of Youth Counselling.	*Communication Self‐Efficacy* Counselling Self‐Estimate (COSE) Inventory. this is a 37‐item questionnaire. *Coping Style* An instrument of 18 items to measure problem‐focused and emotional‐focused coping styles. *Ability to cope* Ten items related to violence risk and safety management, communication and attitude	Before and after the 8‐session training programme. Then observational assessment 3 months after the completion	Mann‐Whitney *U*‐test was used to compare mean differences in the ability to cope with violence between the experimental and control groups AVONA to analyse the variables of communication self‐eefficacy and problem‐focused and emotion‐focused coping styles for inter‐group differences, degree of intra‐group changes and interaction effects
Martinez, 2017	West Coast USA 15 undergraduate Nursing students	Pre‐post test A simulated scenario using a standardized patient (SP) behaving as an agitated psychiatric patient was developed for undergraduate nursing students enrolled in a psychiatric nursing course	Duration of intervention: 4 h Follow‐up: 6 weeks	The Managing Workplace Violence with Evidence‐Based Interventions (MWVEBI) presentation covered topics about WPV, how it affects nurses and nursing students, learning theories.	Mental Health Nursing Clinical Confidence Scale (MHNCCS) was used. The scale was converted to a Likert‐type survey format to be completed by participants The facilitator also designed a 13‐item knowledge assessment questionnaire to assess participants know edge about WPV pre‐ and post‐simulation.	Participants completed the pre‐assessment survey before the simulation, and post‐assessment surveys were completed 6 weeks after the simulation	The yielded results were statistically analysed with SPSS version 22 using paired *t*‐tests
Murray, 2020	Mid‐West USA 20 Student Nurses	Pre‐post test Pilot study 30‐min education module on Incivility	10 weeks + pre and post testing	Bandura's Social Cognitive Theory (1986)	Schwarzer and Jerusalem's 10 item Generalized Self‐Efficacy (1995) Confidence Questionnaire was used (Mallette, Duff, McPhee, Pollex, & Wood, 2011). This questionnaire is domain‐specific to horizontal violence. It is a 3‐item, 100‐point scale measuring the ability to recognize and respond to horizontal violence and modify responses to horizontal violence as the situation change	2 self‐report questionnaires	Using SPSS. Kolmogorov–Smirnov test of normality; a paired‐samples *t*‐test and Wilcoxon Signed Rank Test were used
Nau et al., 2009	Germany 63 nursing students	Quasi experimental longitudinal pre‐ and post‐test study Educational Training course in preventing and managing aggressive patient behaviour	3‐day training course used before training, immediately after the training, and after 2 weeks in clinical placement	Bandura's Self‐efficacy Theory (1993) Dunn et al (2007) Self‐efficacy and locus of control theory Thackery (1987) Clinical confidence	Thackrey's confidence in coping with patient aggression scale	Self‐reported survey	Statistical analysis using Wilcoxon‐test to compare results across the three‐time intervals were done
Nau et al., 2010	Germany 78 Nursing Students	Pre‐test–post‐test within‐and‐between‐groups design (cross sectional and longitudinal two groups before and after design)	Implementation of a 24‐session in 1 week of an aggression management programme	Development of knowledge and competence theories (Arends 2006, Arnold & Siebert 1999; Petersen 2000).	De‐escalating Aggressive Behaviour Scale (DABS), not knowing whether the videos had been recorded before or after the training	De‐escalation experts from three German‐speaking countries evaluated 156 video scenes (of the students faced with a scenario after the training) using the Deescalating Aggressive Behaviour Scale (DABS), not knowing whether the videos had been recorded before or after the training.	Statistical analysis—Wilcoxon and Mann–Whitney *U*‐test was done to compare groups.
Sanner‐Stiehr, 2018	Mid‐West USA 129 nursing students at pre‐post. 119 nursing students at 3 months	Longitudinal quasi experimental A cognitive rehearsal intervention to increase self‐efficacy to respond to disruptive behaviours	Pre‐post‐test at first time point and then 3‐month follow‐up	Social Cognitive Theory Kirkpatric Model (2006) and Bandura Self‐ efficacy (1997)	Self‐efficacy to respond to disruptive behaviours questionnaire (SERDBS) Bandura, 1997	Paper questionnaire at the time pre‐post‐test, electronic questionnaire at 3 months	SPSS, paired samples *t*‐tests and descriptive statistics
Sanner‐Stiehr & Ward‐Smith, 2015	The USA 88 Nursing students	Experiments Randomized Cluster design. Cognitive Behaviour Therapy intervention linked to Social Cognitive Theory framework that resulted in a 1‐h training session	1 h for the training, then pre‐post test conducted and then followed up again 3 months later	Social Cognitive Theory Bandura (1989)	Adaptation of the Scale to address Disruptive Physician Behaviour Scale SADBS‐Revised	Paper questionnaires at pre‐post, but not stated what the 3‐month version was 3, pre‐post‐test in class, then 3 months later	SPSS, paired samples *t*‐tests, descriptive statistics
Schaefer, 2014	Upper West USA Total of 71 nursing students 35 nursing students in the intervention group 36 nursing students in the control group	Mixed method, two group randomly assigned interventional trial Recognition of negative behaviour tool students exposed to a video with content reflecting 12 different negative behaviours (belittle, exasperated sigh, correct in front of patient, talk behind back, grab equipment, non‐verbal gestures, grab arm, ignore/withhold info, rudeness, refuse to help, sarcasm and ignore)	Data were collected 1 week after intervention	Conceptual Model for fostering Civility in Nursing Education (Adapted for Nursing Practice) Clark and Olender 2010	Recognition of Negative behaviour Tool, based on Iennaco (2013)	Paper questionnaire, one point in time immediate post video	SPSS, paired samples *t*‐tests and descriptive statistics
Stephen et al., 2022	Canada 24 undergraduate nursing students	Mixed methods study with RCT Pre and post‐test measures and Focus groups Intervention was a virtual mental health crisis situation conducted via Zoom technology	two focus groups but time frame not specified 4 participants in first FG, 2 in the second FG	Plus Delta Simulation Debriefing Model and Critical Incident Stress Debriefing Framework	Scales used included: Survey for demographic information; Workplace violence awareness and knowledge measures (Brann & Hartley 2017) Measurement of attitudes towards simulation (Allen 1986). Community attitudes to mental Health scale (Taylor & Dear 1981)	Not stated specifically, but after the simulation activity	Descriptive statistics used to summarize the data using SPSS Thematic analysis of themes arising from focus groups
Tecza et al., 2018	USA 314 Nursing Students to develop unit specific interventions 410 nursing students surveyed after intervention administration	Pre‐post (non‐equivalent) A list of WPV interventions was designed based on the pre‐test survey. Mandatory education for nursing staff about WPV. Educational blitzes sessions include strategies for working with students, video examples WPV towards students from nursing staff	Unclear August 2015 to February 2016, and post intervention data were collected August 2016 to February 2017	Duffy's Quality Caring Model	The clinical instructors pursued evidence‐based clarification of this issue and developed a valid and reliable research tool entitled Nursing Student Perception of Civil and Uncivil Behaviours (NSPCUB) in the Clinical Learning Environment Survey. The instrument consists of 12 items, with each researcher‐generated construct (mutual respect, guided participation and student centeredness) having 4 items	Before and after the intervention. The preintervention data collection was performed from August 2015 to February 2016, and post‐intervention data were collected August from 2016 to February 2017	The preintervention and postintervention means were compared using an independent sample *t*‐test.
Zeller et al., 2006	Switzerland Total of 117 nursing students 57 nursing students in the intervention group 60 nursing students in the control group	Quasi experimental pre‐test post‐test design with a non‐equivalent control group Asked 4 questions, regarding frequency of exposure to violence, whilst working (I presume this is placement) 23‐item survey (Beech, 1999). Thackery (1987) measures of feelings when exposed to aggression	24 sessions of 50 min each over 4 consecutive days pre‐test for students 2 days prior to training, not stated if all students in intervention or control. Post‐test with IG, first post‐test immediately after training and second post‐test 3 months after training. CG had the same time frame	Bandura's Theory on Self‐Efficacy (1997)	Data collected included demographic, level of education, and experiences of dealing with patient aggression One dimensional instrument measuring feeling when dealing with aggressive patients 23‐item instruments by Beech (1999) measuring, attitude, competence, expectations, and behaviour towards patients with aggressive behaviours	Two days prior to training, immediately after training and 3 months after training for both groups	Descriptive and inferential statistics Chi square tests, SPSS Chi Squared, tests and Univariate analysis of variance with repeated measures—SPSS programme

### 3.8 Data synthesis

Due to the heterogeneity in the interventions and outcomes, study findings were unable to be pooled statistically. Since meta‐analysis was not possible, data were extracted and reported in tabulated synthesis form without meta‐analysis (Tables 1, 4, and 5). Study outcomes were identified as either patient‐on‐student or staff‐on‐student and statistical analysis results are included to support synthesis findings.

## 4 RESULTS

### 4.1 Study selection

The comprehensive literature search amassed *N* = 403 studies. After removal of the duplicates (*n* = 144), *n* = 263 studies were screened for relevance to the review question. Following the screening, the full text of *N* = 23 studies was retrieved for in‐depth eligibility screening (see Figure 1, PRISMA). Ultimately, 13 studies were included in the review.

### 4.2 Summary of included studies

The thirteen studies included in this review explored the effectiveness of various interventions that aimed to prepare or support RNS in dealing with WPV events in the clinical practice environment. No study provided empirical evidence of the effectiveness of interventions to reduce reported incidences of WPV. Whilst empirical evidence from the many measurement tools used in this group of studies is available, the heterogeneity of interventions, measurement tools used and outcomes sought across the reviewed studies prevented a meta‐analysis from being conducted. Most studies employed interventions grounded in specific theories to explore outcomes, including confidence, knowledge, attitudes, coping, and competence for managing WPV. The authors presented the immediate short‐term outcomes, specifically those occurring within the first 3 months post‐intervention, as most of the included studies' durations were short‐term. Most studies used validated and established tools such as a scale measuring confidence in coping with patient aggression or something similar for data collection. Studies in the summary table have been allocated to either the “patient‐on‐student” group or a “staff‐on‐student” group based on the perpetrator focus, reported aims and outcomes of each study (Table 4).

### 4.3 Characteristics of studies

One of the 13 studies was an RCT (Sanner‐Stiehr & Ward‐Smith, 2015), and the remainder (*n* = 10) were quasi‐experimental pre‐test‐post‐test studies, including Cherian & Sharma, 2019; Stephen et al., 2022; Sanner‐Stiehr, 2018; Nau et al., 2010; Nau et al., 2009; Murray, 2020; Martinez, 2017; Beech & Leather, 2003; Schaefer, 2014; Zeller et al., 2006; and (*n* = 2) before and after studies: Jeong & Lee, 2020; Tecza et al., 2018. Six studies were conducted in the United States (Martinez, 2017;Murray, 2020; Sanner‐Stiehr, 2018; Sanner‐Stiehr & Ward‐Smith, 2015; Schaefer, 2014; Tecza et al., 2018), two studies from Germany (Nau et al., 2009; Nau et al., 2020) and one study in each of the following countries: Canada (Stephen et al., 2022), India (Cherian & Sharma, 2019), South Korea (Jeong & Lee, 2020), the United Kingdom (Beech & Leather, 2003) and Switzerland (Zeller et al., 2006). The number of participants ranged from 15 to 314.

Predominantly, the context of WPV explored was patient‐on‐staff violence, while five studies (Murray, 2020; Sanner‐Stiehr, 2018; Sanner‐Stiehr & Ward‐Smith, 2015; Schaefer, 2014; Tecza et al., 2018) examined the impact of educational interventions in the context of staff‐on‐student incivility. The division into these two categories was justified because the differing objectives and outcomes sought by researchers based on the type of perpetrator required separate analyses. The characteristics and findings of these two groups are reported separately (see Table 1).

Educational interventions were the most common intervention and the total duration of educational interventions ranged from 30 min to 4 weeks, with all papers reporting responses to interventions within the short time frame, either immediately post‐test or within 3 months. The types of interventions, delivery methods, time and their effectiveness are presented in Table 5.

Theoretical constructs were mainly used to design and underpin educational interventions, but not the content. Albert Bandura's Social Cognitive Theory was used in six studies (Martinez, 2017; Murray, 2020; Nau et al., 2009; Sanner‐Stiehr, 2018; Sanner‐Stiehr & Ward‐Smith, 2015; Zeller et al., 2006). Due to a range of theoretical underpinnings used, the modality was mixed with didactic style, e‐learning, role play and simulation. The questionnaires used were diverse, even when measuring a single domain. For example, confidence was assessed using three different questionnaires: Confidence in Coping with Patient Aggression Scale (Nau et al., 2009), De‐escalating Aggressive Behaviour Scale (DABS) (Nau et al., 2010) and Mental Health Nursing Clinical Confidence Scale (MHNCCS) (Martinez, 2017). Outcomes were measured at different timepoints, which for the purposes of this review, studies were usually immediately post‐intervention or within 3 months.

The focus and aims of each study were closely related to identified perpetrators of WPV and exerted significant influence on the interventions and measurement tools used with two categories of perpetrators: Patient‐on‐Student and Staff‐on‐Student. A total of eight studies reported interventions related to Patient‐on‐Student (Beech & Leather, 2003; Cherian & Sharma, 2019; Jeong & Lee, 2020; Martinez, 2017; Nau et al., 2009; Nau et al., 2010;Stephen et al., 2022; Zeller et al., 2006) and five studies described the Staff‐on‐Student's interventions (Murray, 2020; Sanner‐Stiehr, 2018; Sanner‐Stiehr & Ward‐Smith, 2015; Schaefer, 2014; Tecza et al., 2018) in this review.

#### 4.3.1 Quality and risk of bias appraisal

One study (Jeong & Lee, 2020) met all the study quality criteria (see Table 3). Six papers scored more than 60%, five quasi‐experimental studies (Jeong & Lee, 2020; Martinez, 2017; Sanner‐Stiehr, 2018; Schaefer, 2014; Zeller et al., 2006) and one randomized controlled study (Sanner‐Stiehr & Ward‐Smith, 2015) (see Table 2). Due to the nature of the interventions, there was a lack of allocation concealment and blinding. The greatest limitation of the quasi‐experimental studies was the lack of a control group (Beech & Leather, 2003; Cherian & Sharma, 2019; Martinez, 2017; Murray, 2020; Nau et al., 2009; Nau et al., 2010; Stephen et al., 2022; Tecza et al., 2018). The JBI revised critical appraisal tool provided a detailed view of the internal validity and potential for risk of each of the studies. Whilst only seven of the 13 studies received more than 50% from the quality appraisal, most were considered moderate to low risk of bias, with three studies at high risk (Martinez, 2017; Schaefer, 2014; Tecza et al., 2018) (Table 3). Significant in the increased risk of bias was related to follow‐up of participants who were lost to the study, lack of control group and singular measurements of constructs.

**TABLE 2:** Critical appraisal results for randomized controlled trials.

Citation	Q1	Q2	Q3	Q4	Q5	Q6	Q7	Q8	Q9	Q10	Q11	Q12	Q13	%
Sanner‐Stiehr & Ward‐Smith, 2015	Y	N	Y	N	U	U	Y	Y	Y	Y	Y	Y	Y	69.2

*Note*: Q1. Was true randomization used for assignment of participants to treatment groups? Q2. Was allocation to treatment groups concealed? Q3. Were treatment groups similar at the baseline? Q4. Were participants blind to treatment assignment? Q5. Were those delivering treatment blind to treatment assignment? Q6. Were outcomes assessors blind to treatment assignment? Q7. Were treatments groups treated identically other than the intervention of interest? Q8. Was follow‐up complete and if not, were differences between groups in terms of their follow‐up adequately described and analysed? Q9. Were participants analysed in the groups to which they were randomized? Q10. Were outcomes measured in the same way for treatment groups? Q11. Were outcomes measured in a reliable way? Q12. Was appropriate statistical analysis used? Q13. Was the trial design appropriate, and any deviations from the standard RCT design (individual randomization, parallel groups) accounted for in the conduct and analysis of the trial?

Abbreviations: N, no; U, unclear; Y, yes.

**TABLE 3:** Critical appraisal results for quasi‐experimental studies.

		Internal validity										Quality and bias outcomes	
		Bias related to:											
												Appraisal for bias outcome	Critical appraisal results
		Domain	Temporal precedence	Selection and allocation	Confounding factors	Administration of intervention/exposure	Assessment, detection and measurement of the outcome			Participant retention	Statistical conclusion validity	JBI revised appraisal	JBI critical appraisal for quasi experimental studies
		Question No.	1	2	3	4	5	6	7	8	9	(Barker et al 2023)	
Study Id	Outcome	Result										Non weighted average.	
Patient on students													
	Knowledge	Time 1	Y	N	Y	Y	Y	Y	U/C	N	Y	7.5	55.50%
Study 1		Time 2								N	Y		
Beech & Leather, 2003	Confidence	Time 1	Y	N	Y	Y	Y	Y	U/C	N	Y		
		Time 2								N	Y		
	Self‐Efficacy	Time 1	Y	N	Y	Y	Y	Y	U/C	N	Y		
		Time 2								N	Y		
	Competence	Time 1	Y	N	Y	Y	Y	Y	U/C	N	Y		
		Time 2								N	Y		
Study 2	Knowledge	Time 1	Y	Y	Y	Y	Y	Y	Y	U/C	Y	8.5	88.80%
Zeller et al., 2006		Time 2								U/C	Y		
	Competence	Time 2	Y	Y	Y	Y	Y	Y	Y	U/C	Y		
		Time 1								U/C	Y		
	Attitudes	Time 1	Y	Y	Y	Y	Y	Y	Y	U/C	Y		
		Time 2								U/C	Y		
Study 3	Confidence	Time 1	Y	N	Y	Y	N	Y	N	U/C	Y	6.5	33.30%
Nau et al., 2009		Time 2								U/C	Y		
	Self‐Efficacy	Time 1	Y	N	Y	Y	N	Y	N	U/C	Y		
		Time 2								U/C	Y		
	Competence	Time 1	Y	N	Y	Y	N	Y	N	U/C	Y		
		Time 2								U/C	Y		
Study 4	Knowledge	Time 1	Y	N	Y	Y	Y	Y	U/C	U/C	Y	7	55.50%
Nau et al., 2010		Time 2					Y	Y	U/C	U/C	Y		
	Competence	Time 1	Y	N	Y	Y	Y	Y	U/C	U/C	Y		
		Time 2					Y	Y	U/C	U/C	Y		
Study 5	Knowledge	Time 1	Y	N	Y	Y	N	Y	Y	N	Y	5.6	66.60%
Martinez, 2017		Time 2								N	Y		
	Confidence	Time 1	Y	N	Y	Y	N	Y	Y	N	Y		
		Time 2								N	Y		
	Competence	Time 1	U/C	N	Y	Y	N	U/C	U/C	N	Y		
		Time 2								N	Y		
Study 6	Knowledge	Time 1	Y	N	Y	N	Y	Y	Y	U/C	Y	7.5	22.20%
Cherian & Sharma, 2019		Time 2								U/C	Y		
	Confidence	Time 1	Y	N	Y	N	Y	Y	Y	U/C	Y		
		Time 2								U/C	Y		
Study 7 Jeong & Lee, 2020	Self‐Efficacy	Time 1	Y	Y	Y	Y	N	Y	Y	N	Y	7	100%
		Time 2								N	Y		
	Competence	Time 1	Y	Y	Y	Y	N	Y	Y	N	Y		
		Time 2								N	Y		
Study 8 Stepen et al., 2022	Knowledge	Time 1	U/C	Y	Y	Y	Y	Y	Y	Y	Y	8.5	22.20%
		Time 2								Y	Y		
Staff on student													
Study 9	Knowledge	Time 1	Y	N	Y	Y	Post‐test only	Y	Y			6	66.60%
Schaefer, 2014		Time 2								U/C	U/C		
	Knowledge	Time 1	Y	N	Y	Y	N	Y	Y	Y	Y		88.80%
Study 10		Time 2								Y	Y	7	
Sanner‐Stiehr, 2018	Self‐Efficacy	Time 1	Y	N	Y	Y	N	Y	Y	Y	Y		
		Time 2								Y	Y		
	
Study 11		Time 1	Y	N	Y	N	N	Y	Y	U/C	U/C	5	44.40%
Tecza et al., 2018	Reduction in Lateral Violence	Time 2								U/C	U/C		
Study 12	Confidence	Time 1	Y	N	Y	Y	Y	Y	U/C	Y	Y		
Murray, 2020		Time 2								Y	Y	7.5	33.30%
	Self‐Efficacy	Time 1	Y	N	Y	Y	Y	Y	U/C	Y	Y		
										Y	Y		

*Note*: (JBI Quasi experimental studies and Revised JBI Quasi Experimental studies for Bias). Quasi experimental biases appraisal. Yes(Y) = 1, No(N) = 0, Unclear (U/C) = .5. Total score is non‐weighted Average /9. >8/9 = Low Risk, 7/9 = medium risk, <6.5 = High risk.

### 4.4 Outcomes

Across all studies, the four most common outcomes measured were knowledge, confidence, self‐efficacy and competence, where significant improvements were found. Interventional content, duration and effectiveness measurement varied between studies, as presented in Table 4. In terms of content, many constructs were examined across the studies, but no study tested all outcomes.

**TABLE 4:** Summary of findings.

Author(s)	Intervention		Outcomes	Summary of findings
Patient‐on‐student	Content	Mode	Short‐term outcomes (up to 3 months post‐intervention)	
Beech & Leather, 2003	Knowledge of psychological theories and models, attitudes, increasing self‐esteem, and breakaway skills	Face‐to‐face	Investigator‐developed questionnaire measuring self‐reported attitudes, estimated behavioural competence, confidence, and belief in myths. The domains of this questionnaire were: Maintaining personal safety Prediction and prevention Practical ability Self‐respect and staff rights Provocative approach. *Knowledge*: Theories and Models, Incidences, risk factors, prediction and prevention *Confidence*: in ability *Competence*/*Skills*: therapeutic approach/stance, interpersonal skills, verbal, and non‐verbal skills	Evidence that training in workplace violence can be effective in producing knowledge, behavioural, attitude, and confidence changes. The changes identified in this study included areas of risk factor identification and specific factors of: maintaining personal safety, Prediction and prevention, Practicable ability, Self‐respect and staff rights and provocative approaches. Varying levels of significance were identified. Number of risk Factors Identified Scenario 1‐ pre‐test 4.18, post‐test 4.89 Scenario 2‐ pre‐test 4.41, post‐test 5.00. Intervention Impact: Factor 1—Significant difference at post Day 1 *p* < .0010 and 3 months *p* < .001 Factor 2—Significant difference at post Day1 *p* < .001 and 3 months *p* < .001 Factor 3—Signficant difference at post‐Day 1 < .001 and 3 months *p* < .001 Factor 4—Significant difference at post‐Day 1 *p* = .002 and 3 months *p* = .001 Factor 5—Significant difference at post‐Day 1 *p* < .001 and 3 months *p* < .001
Cherian & Sharma, 2019	Training programme on aggression management	Programme presented via lectures, presentations, and role playing	*Knowledge*: of aggression management *Confidence*: Investigator‐developed questionnaire Competency	There was a statistically significant improvement in knowledge (*t* = 13.2, *p* < .01): Pretest mean = 11.24 (SD 2.62), post‐test mean = 19.36 (SD 4.31) There was a statistically significant improvement in confidence (*t* = 5.58, *p* < .01): pre‐test mean = 26.05 (SD 8.02), post‐test mean = 32.85 (SD 7.69) The results for knowledge and confidence were extrapolated to competency
Jeong & Lee, 2020	Communication, assertiveness training and self‐defence	Multimodal: face‐to‐face, videos, role play and practical scenarios	*Self*‐*efficacy*: Counselling Self‐Estimate (COSE) Inventory. Communication self‐efficacy *Coping*: Investigator‐developed questionnaire to assess for problem‐focused and emotion‐focused coping styles; and ability to cope with violence	There was no statistically significant difference in communication self‐efficacy between intervention (mean = 138.23, SD 8.01) and control groups (mean = 139.17, SD 9.72) The intervention group had significantly better scores in problem‐focused coping style compared to control group (*p* < .01): intervention mean = 47.59 (SD 4.71), control group 38.87 (SD 4.93) There was no statistically significant difference in emotion‐focused coping styles between intervention (mean = 14.82, SD 3.29) and control groups (mean = 18.35, SD 3.11) The intervention group had significantly higher score in their ability to cope with violence than the control group (*p* < .01): Intervention mean = 10.27 (SD 2.10), control mean = 5.7 (SD 2.1)
Martinez, 2017	Types of workplace violence and how they impact nurses, incident trends and evidence‐based interventions to prevent and manage violence	Simulation	*Knowledge*: of WPV *Confidence*: To recognize aggression, Mental Health Nursing Clinical Confidence Scale (MHNCCS) *Competence*: to de‐escalate aggressive situation	Increase of knowledge range from 6% to 53% There was a statistically significant improvement in confidence (*t* = 5.68; *p* < .01): Pre‐test mean = 30.15 (SD 7.88), post‐test mea*n* = 45.13 (SD 6.91) Competence not described
Nau et al., 2009	Causes of aggression, defusing risks of aggression, recognizing escalating situations, respecting the patient, depersonalizing, interpreting the situation related to nursing interventions that failed and reflecting on safety issues and well‐being.	Multi‐modal: face‐to‐face, group discussion, multimedia and reflection	*Confidence*: Confidence In Coping with Patient Aggression Scale *Knowledge*: Management of aggressive patients	There was a statistically significant improvement in confidence (Wilcoxon test, *p* < .001): Pre‐test mean = 2.51 (no SD reported), post‐test mean = 3.69 (no SD reported) Ability to handle physical aggression decreased over time *p* < .048 Perceived safety in WPV event increased *p* < .033 Knowledge development 10/63 participant reported improved knowledge/understanding of what to do in WPV encounter.
Nau et al., 2010	Twenty‐four training sessions within 1 week. Training included aspects of: prevention, assessment of occurrence, dealing with the patient, and coping and after care.	Student responses to two scenarios pre training and post training Training was 24 sessions within a 1‐week time frame Data were collected via video taping.	*Confidence*: De‐escalating Aggressive Behaviour Scale (DABS) *Competency*: performance de‐escalating aggressive situations. *Knowledge*: Influence of intervention on learning progression	Post interventions students performed significantly better DABS (Mann–Whitney *U*‐test *p* < .01): Scenario A DABS pre‐test median = 2.5, post‐test median = 3.7 (Mann–Whitney *U*‐Test *p* < .01). Scenario B DABS pre‐test median = 3.01, post‐test median = 3.61 post‐test Competency in de‐escalating aggression Inconsistent results regarding learning progression. Level of education significant (*p* = .02). Age, duration of nursing education not significant
Stephen et al., 2022	Awareness and responding to mental health deterioration	Simulation	*Knowledge*: Workplace violence awareness and knowledge measures (Brann & Hartley 2017) *Attitudes*: Measurement of attitudes towards simulation (Allen 1986); Community attitudes to mental Health scale (Taylor & Dear 1981)	Insufficient participants to detect effect. Knowledge scores were reported as overall value of the information provided in the intervention but were not significant due to small numbers of participants. Pre‐test mean = 29.4 (95% CI 27.7–31.2), post‐test mean = 31.5 (95% CI 29.7–33.29). Unclear evidence to support the effectiveness of educational intervention as measured by attitudes
Zeller et al., 2006	Types of aggression, forms and causes of violence, dealing with ones own aggression, theory about aggression behaviours, handling situations/conflict, de‐escalation strategies, communication instruments, behaviour after an aggressive event, safety and prevention in the work place, liberation and defence techniques, and practice of communication techniques.	Multi‐modal: face‐to‐face, role play, multimedia, reflection	*Knowledge*: Dealing with aggressive patients. *Confidence*: Confidence In Coping with Patient Aggression Scale *Attitudes*/*Competence*: Attitude and behaviour towards aggressive patients Questionnaire from Beech and Leather (2003) measuring self‐reported attitudes *Self*‐*efficacy*: confidence, and belief in myths.	The impacts of the educational intervention were unclear and inconsistent. Feelings of competence and security stated significantly improved *p* < .001 Feeling seriously /physically Threatened (once only): Experimental Group (EG) 32%, Control Group (CG) 22%. More than once; EG 11%, CG 10% Q 3: Physical Attacks experienced: EG 32%, CG 22%. Q4: Verbal attacks, EG 72%, CG 77%. Overall, no significant difference (p value not stated) Expectations or competence measured with Thackery instrument (1989), 7 items highly significant (*p* value .05). Beech instrument (1999) 6/23 items significant (p.y 0.001) Knowledge and attitude did not show significant improvements.
Staff‐on‐student				
Murray, 2020	Identifying and addressing incivility in the workplace	Multimodal: face‐to‐face, case studies, simulation, group discussion, and role play	*Self*‐*efficacy*: Generalized Self Efficacy Scale Confidence: Confidence Questionnaire (recognize and respond to horizontal violence and modify responses to horizontal violence as the situation change)	There was a statistically significant improvement in self‐efficacy (*p* < .01) Pre‐test mean = 3.03 (SD 0.35), Post‐test man = 3.38 (SD 0.30) It was reporter that there was a statistically significant improvement in self‐confidence scores in recognizing and addressing uncivil behaviours (*p* < .01) Paired *t*‐test were used for the parametric data GSE scores, and Wilcoxon signed rank *t*‐test was used for nonparametric data—Confidence Questionnaire scores. Self‐efficacy—GSE score were statistically significantly pre‐test(M = 3.03, SD = 0.35) and post‐test (M = 3.38, SD =0.30) with an eta squared effect of (*r* = .55) Medium. Confidence—a statistically significant increase in confidence measured after completion of the education intervention was found—z = −3.741, 0 < .001 with a medium effect size (*r* = 0.59)
Sanner‐Stiehr, 2018	Responding to disruptive behaviours	Classroom	*Self*‐*efficacy*: Intervention to improve RNS's self‐efficacy to respond effectively to disruptive behaviours. Self‐efficacy to Respond to Disruptive Behaviours Questionnaire	It was reported that there was an overall improvement in self‐efficacy scores It was reported that the educational intervention promoted self‐efficacy in dealing with encounters of workplace violence Pre and post‐test mean scores were measured with paired t‐tests with CI of 95% and found scores of 9.41, 9.45, and 9.27/100 across measures of self‐efficacy, knowledge and motivation, (*p* < .001). At 3 months self‐efficacy and knowledge was not significantly changed (*p* < .002; *p* < .020)
Sanner‐Stiehr & Ward‐Smith, 2015	To address disruptive behaviour a cognitive rehearsal intervention that targeted cognitive and affective domains was delivered to participants	Role‐play	*Self*‐*efficacy*: Disruptive Physician Behaviour Scale SADBS‐Revised‐measured self‐efficacy to respond to lateral violence Measured responses to verbal abuse, non‐verbal inuendo, gossiping, scapegoating, undermining, refusal to help, sabotage, failure to respect privacy, broken confidences, withholding of information	This study employed an experimental, single‐blinded time series, randomized cluster design. Paired t‐tests were used to identify significant changes between pre‐ and post‐test responses. Descriptive statistics were used for the characteristics of participants. In the Experimental Group (EG), there was statistically significant increases across responses in all 10 instruments used (*p* = .00), power of .95 and effect size of .40. Testing of the EG 3 months post intervention found no significant difference to immediate post intervention results and was considered evidence of a sustained effect of the intervention
Schaefer, 2014	Scenarios of negative behaviours: belittle, exasperated sigh, correct in front of patient, talk behind back, grab equipment, non‐verbal gestures, grab arm, ignore/withhold info, rudeness, refuse to help, sarcasm, ignore	Video	*Knowledge*: recognizing verbal abuse *Self*‐*efficacy*: Recognition of Negative Behaviour Tool	Statistical significance was found in the post‐test finding in the aspect of recognizing non‐verbal abuse. The exasperated sigh by the nurse was found to be statistically significant with the score for the intervention group (M = 1.49), SD = .507; *t* (67) =2.679, *p* = .009 (two‐tailed)
Tecza et al., 2018	A Hospital staff development intervention aimed at reducing incivility and lateral violence towards nursing students. The interventions were diverse and clinical unit specific	Interactive and education formats	*Knowledge*: Measuring Nursing Student Perception of Civil and Uncivil Behaviours in the Clinical Learning Environment.	It was reported that there was an improvement in students' capacity to recognize incivility in the clinical environment. Significant statistical differences were found in the mean scores of 7 of the 12 analysed using *t*‐tests. Significance was considered when a p value of <.05 was found. Reliability was established with a Cronbach's a high score of 0.933,0.880 and 0.863 for the constructs measured.

The reported outcomes included knowledge, confidence, self‐efficacy, competence/skills and attitudes. **Knowledge** was measured by eight studies (Beech & Leather, 2003; Cherian & Sharma, 2019; Martinez, 2017; Nau et al., 2010; Sanner‐Stiehr, 2018; Schaefer, 2014; Stephen et al., 2022; Zeller et al., 2006). **Confidence** was measured by four studies (Beech & Leather, 2003; Cherian & Sharma, 2019; Martinez, 2017; Nau et al., 2009). **Self**‐**efficacy** was measured by six studies (Beech & Leather, 2003; Jeong & Lee, 2020; Murray, 2020; Nau et al., 2009; Sanner‐Stiehr, 2018; Sanner‐Stiehr & Ward‐Smith, 2015). **Competence** was measured by five studies (Beech & Leather, 2003; Jeong & Lee, 2020; Martinez, 2017; Nau et al., 2009; Nau et al., 2010), and **Attitudes** by two studies (Stephen et al., 2022; Zeller et al., 2006). Only one study Tecza et al. (2018) identified the impact of an intervention on the incidence of WPV on the experiences of RNS.

**Knowledge** was measured by eight of the studies, and the results were statistically significant in three studies: Beech & Leather, 2003 (no aggregated data reported); Cherian & Sharma, 2019 (t = 13.2, *p* < 0.01), Pre‐test mean = 11.24 (sd 2.62), post‐test mean = 19.36 (sd 4.31) and Sanner‐Stiehr, 2018 (*p* < .000). However, knowledge was not statistically significant in five studies: Zeller et al., 2006; Nau et al., 2010; Martinez, 2017; Stephen et al., 2022; Schaefer, 2014.

**Confidence** was found to be significant in all five studies measuring this outcome: Beech & Leather, 2003 (no aggregated data reported); Cherian & Sharma, 2019 (t = 5.58, *p* < .01), pre‐test mean = 26.05 (sd 8.02), post‐test mean = 32.85 (sd 7.69); Martinez, 2017 (t = 5.58, *p* < .01), pre‐test mean = 30.15 (sd 7.88), post‐test mean = 45.13 (sd 6.91); Nau et al., 2009 (no sd reported), pre‐test mean = 2.51, post‐test mean = 3.69 (no sd reported); **Self**‐**efficacy** was examined by six studies with five finding significant improvement: Beech & Leather, 2003 (no data reduction only itemized results); Nau et al., 2009, Significant t2 & t3 (*p* < .001); Sanner‐Stiehr & Ward‐Smith, 2015 (*p* = .09); Sanner‐Stiehr, 2018 and Murray, 2020 (p,.000). **Competence** was found to be significantly improved at 3 months (*p* = .001) by Nau et al., 2009 and Zeller et al., 2006. However, not all studies found significant improvements, with Zeller et al. (2006), Stephen et al. (2022) and Schaefer (2014) having a number of variables where no significant improvements were found in Tables 4 and 5.

**TABLE 5:** Detailed summary of findings.

Papers	Interventions					Core constructs and findings significance				
	Content Number of participants (n=)	Delivery schedule	Delivery mode	Time between intervention and post measurement t2	Time of last measurement t3	Knowledge	Confidence	Self‐efficacy	Competence/Skills	Attitudes
Patient‐on‐student										
Beech & Leather, 2003	Knowledge of psychological theories and models, attitudes, increasing self‐esteem, breakaway skills (*n* = 243)	3 days 3 cohorts End of 1st year All within 2 weeks	Face‐to‐face. Self‐awareness activities, Role playing, multi‐ media, de briefing	End day of the intervention	3 months after delivery	Risk factors identified. Prediction and prevention Significant (no data reduction, itemized p values provided)	Self‐respect and rights Significant (no data reduction, itemized *p* values provided)	Self‐esteem and confidence including, own safety and interacting non‐provocatively Significant (no data reduction, itemized p values provided)	Practical ability Decreased score post and t 3 months. Not significant	Attitudes to safety Significant (no data reduction, itemized p values provided
Cherian & Sharma, 2019	To investigate the cogency of aggression management training programme on competency of nursing students (*n* = 71)	Definition Characteristics, types, causes, observation communication and therapeutic relationship prevention and management	Lectures, power points, and role playing	Post intervention testing within 1 week of intervention completion	No follow‐up conducted	Knowledge of aggression management increased post intervention Significant (*p* = .00)	Confidence in aggression management improved post intervention Significant (*p* = .00)			
Jeong & Lee, 2020	Communication, assertiveness training, and self‐defence (*n* = 45, EG = 22, CG = 23)	16 h Eight sessions 2 sessions per week over 4 weeks	Multimodal: face‐to‐face, videos, role play, practical scenarios	Post‐test observation of simulation immediately on completion	Simulation was repeated at 3 months post completion of intervention			Communication self‐efficacy—no significant difference (*p* = 0.437) Problem focus‐coping—significant (*p* < .001) Emotion focused coping not significant (*p* = .156)	Ability to cope with violence Significant (*p* < .001)	
Martinez, 2017	Types of workplace violence and how they impact nurses, incident trends, and evidence‐based interventions to prevent and manage violence (*n* = 15)	4 h Written educational materials concerning the assault cycle. 2 rehearsal sessions	Blended‐F2F and online Simulation	5‐min recorded simulation feedback provided −2 min immediately following the simulation	Not repeated	Post knowledge of five questions (questions not stated) Unclear significance although improvement stated in “most “of the questions	Measured by MHNCCS Confidence but in what not stated. Statistically significant (*p* < .0001)		Measured as recognition of signs of aggression. Statistical significance not displayed but improvements stated	
Nau et al., 2009	Causes of aggression, defusing risks of aggression, recognizing escalating situations, respecting the patient, depersonalizing, interpreting the situation related to nursing interventions that failed and reflecting on safety issues and well‐being (*n* = 63)	3 days 8 sessions /day 45 min per session	Multi‐modal: face‐to‐face, group discussion, multimedia, reflection	Immediately following Intervention	Between 4–8 weeks following the intervention		Confidence in coping Significant t2 and t3 (*p* < .001)	Perceived safety in dealing with aggressive Patient. Significant at t2 and t3 (*p* = .033)	Perceived ability to handle physical aggression. Significant at t2 (p not stated) decreased at t3 (*p* < .048)	
Nau et al., 2010	Training included aspects of prevention, assessment of occurrence, dealing with the patient, coping and after care (*n* = 78)	24 training sessions delivered within 1 week Sessions up to 1 h in length	Teaching sessions face to face including 2 scenarios.	Video recording of scenario recordings assessed using DABS scale at completion of training	Not repeated	Learning progression results are unclear and varied. No overall result No influence of age (*p* = .76, pervious nursing education (*p* = .30) Not significant			Performance improvements following training. Significant across all measurements t2(*p* < .001)	
Stephen et al., 2022	Awareness and responding to mental health deterioration (*n* = 24)	1 session of simulated learning prior to assessment	Virtual Simulation 10–15 min duration followed by debrief 10–20 min	Post‐test immediately following scenario	Not repeated	The main area of interest in this study was knowledge development but there was insufficient sample for differences to be detected Not significant				
Zeller et al., 2006	Types of aggression, forms and causes of violence, dealing with one's own aggression, theory about aggression behaviours, handling situations/conflict, de‐escalation strategies, communication instruments, behaviour after an aggressive event, safety and prevention in the workplace, liberation and defence techniques, practice of communication techniques. (*n* = 117, EG = 57, CG = 60)	24 × 50‐min sessions over 4 days Varied between first and fourth year of training	Multi‐modal: face‐to‐face, role play, multi‐media reflection	Immediately following intervention	3 months post intervention	Knowledge Not Significant	Feelings of security Improved but not significant		Competence Highly significant immediately following and at 3 months (*p* = .001)	Attitude/feelings of security Not Significant
Staff‐on‐students										
Murray, 2020	Identifying and addressing incivility in the workplace (*n* = 20)	30‐min education module on incivility for 10 weeks. Total of 300 min	Multimodal face‐to‐face, case studies, simulation, group discussion and role play	Timing of post intervention testing not stated	No follow‐up testing (pilot study)		Improved confidence in addressing incivility and recognizing uncivil behaviours. Significant (*p* = .001)	Improved self‐efficacy to recognize incivility. Significant (*p* < .000)		
Sanner‐Stiehr & Ward‐Smith, 2015	To address disruptive behaviour a cognitive rehearsal intervention that targeted cognitive and affective domains was delivered to participants. (*n* = 88)	1 h CBT training session	Role‐play cognitive behavioural rehearsal in self‐ efficacy to respond to lateral violence uses education, data collected via survey pre and post intervention demonstration, rehearsal, feedback, debriefing	Immediately on completion of the training	Repeated 3 months later			Perceived self‐efficacy to respond to lateral violence Significant difference t1 and t2, no significant difference between t2 and t3 indicating a sustained effect *p* = .09		
Sanner‐Stiehr, 2018	Responding to disruptive behaviours self‐efficacy to respond to behaviours (*n* = 129)	Education 30 min Intervention delivered during class time and included‐education session, role modelling, cognitive rehearsal, debriefing	Classroom activity	Immediately following training	Follow‐up survey at 3 months post‐intervention	Items measured knowledge and motivation Knowledge significantly improved post intervention (p values for individual items provided, *p* < .000) t2 and t3 CI 95%		Self‐efficacy to respond/strategies to disruptive behaviours Significant improvement of items in the survey (multiple p values) provided t2 and t3 CI 95%		
Schafer, 2014	Scenarios of negative behaviours: belittle, exasperated sigh, correct in front of patient, talk behind back, grab equipment, non‐verbal gestures, grab arm, ignore/withhold info, rudeness, refuse to help, sarcasm, ignore. (*n* = 71) EG =35,CG =36)	1 h training program on recognition and reporting of negative behaviour	Class based training programme 6 short Videos	Post‐test data collected immediately on completion of the intervention	No follow‐up data collected	Intervention group found increased recognizing non‐verbal abuse, exasperated sigh Significant. (*p* = .009) EG and CG both able to recognize verbal and physical abuse Not significant				
Tecza et al., 2018	This study aimed at reducing lateral violence experienced by RNS and perpetrated by nurses. The Intervention session was conducted during Staff development session. (*n* = 314)	A video during hospital education structured event I interactive and 1 educational intervention	Varied intervention designed by Unit sections. Must include a video and classroom activity	Post‐intervention but time frame unclear	No follow‐up recorded.					This study examined the effect of an education session provided to RNs on RNS. It found that 7 of 12 items had a significant improvement2 overall t1 = 3.2, t2 = 3.6, *p* = .001

#### 4.4.1 Primary outcome: WPV Incidence

Of the 13 studies reviewed, only one study (Tecza et al., 2018) used the incidence of WPV as a measure of intervention effectiveness. Tecza et al.'s (2018) approach to the problem of WPV experienced by RNS was an intervention directed at unit and hospital‐wide staff rather than RNS and measured the effect of the intervention as RNS experiences of WPV and found a significant decrease in WPV experiences (*p* = .001). The omission of incidence in the majority of papers as a measure of effectiveness reflects the complex inter‐relationship between knowledge, skills, confidence and recognition of WPV and an underlying expected increase in reporting of WPV events as these constructs are developed by RNS. While most studies aimed to equip RNS with the skills to recognize, prevent and manage WPV, the incidence of WPV is a higher‐level outcome and was not considered by the authors of most studies as an immediate measure of the effectiveness of a specific intervention.

#### 4.4.2 Secondary outcomes

##### Patient‐on‐student WPV

Eight studies explored interventions related to patient on student WPV (Beech & Leather, 2003; Cherian & Sharma, 2019; Jeong & Lee, 2020; Martinez, 2017; Nau et al., 2009; Nau et al., 2010; Stephen et al., 2022; Zeller et al., 2006). Interventions developed were diverse and aimed to improve RNS knowledge, confidence, coping, competence and self‐efficacy related to patient on student WPV, with no single study exploring all constructs. Results for each of the constructs are mixed, with some studies finding high levels of significance and others finding unclear or not statistically significant results.

##### Knowledge

Seven studies (Beech & Leather, 2003; Cherian & Sharma, 2019; Martinez, 2017; Nau et al., 2009; Nau et al., 2010; Stephen et al., 2022; Zeller et al., 2006) assessed the impact of educational interventions on knowledge. Of the seven studies which measured this construct, four found significant improvement (Beech & Leather, 2003; Cherian & Sharma, 2019; Martinez, 2017; Nau et al., 2009; Stephen et al., 2022) and two found no difference (Nau et al., 2010; Zeller et al., 2006) or had insufficient data (Stephen et al., 2022). It was evident where statistical significance was found, intensive management programmes that presented face‐to‐face or incorporated role‐playing or simulated scenarios had the capacity to improve knowledge and potential competence. However, not all interventions successfully increased knowledge despite prolonged engagement, such as Nau et al.'s (2010) intervention that extended over 24 sessions.

##### Confidence

Four studies measured confidence (Cherian & Sharma, 2019; Martinez, 2017; Nau et al., 2009; Nau et al., 2010). From Nau et al. (2009) multi‐modal group discussion and scenario reflections, which found significant improvements in registered nursing students' confidence in managing patient aggression and de‐escalation (Wilcoxon test, *p* < .001), pre‐test mean = 2.51 (no SD reported), post‐test mean = 3.69 (no SD reported), to Martinez (2017) statistically significant simulations (t = 5.68; *p* < .01), pre‐test mean = 30.15 (SD 7.88), post‐test mean = 45.13 (SD 6.91) confidence was considered an important aspect of managing WPV by RNS in the patient‐on‐student situation.

##### Self‐efficacy

Surprisingly, in the patient‐on‐student group, only two studies (Jeong & Lee, 2020; Zeller et al., 2006) measured self‐efficacy. The findings from Zeller et al. (2006) are unclear, and Jeong and Lee (2020) findings related to communication self‐efficacy demonstrated no statistically significant difference between intervention and control groups (mean = 138.23, SD 8.01) and control groups (mean = 139.17, SD 9.72). Jeong and Lee (2020) explored coping from a problem or emotion‐coping focus and found a significant difference in coping skills in problem‐focused scenarios but not emotion‐focused ones (*p* < .01): intervention mean = 47.59 (SD 4.71), control group 38.87 (SD 4.93).

##### Competency

Four studies (Beech & Leather, 2003; Martinez, 2017; Nau et al., 2010; Zeller et al., 2006) identified competency as an essential measure of the success of an intervention. Zeller et al. (2006) reported statistically significant results, expectations, or competence measured with the Thackery instrument (1989), with seven items statistically significant (p‐value .05).

##### Other (attitudes)

Beech and Leather (2003) measured the impact of an educational intervention on self‐reported attitudes, estimated behavioural competence, confidence and belief in “myths” using one questionnaire. Results showed that there were no statistically significant improvements in these outcomes. One other study assessed attitudes, which showed unclear evidence to support the effectiveness of educational intervention (Stephen et al., 2022).

###### Staff‐on‐student WPV

Five studies were included in this category of WPV perpetrator‐focused interventions (Murray, 2020; Sanner‐Stiehr, 2018; Sanner‐Stiehr & Ward‐Smith, 2015; Schaefer, 2014; Tecza et al., 2018), with self‐efficacy and confidence being the most common constructs measured. This categorization recognized the intended focus of the intervention, WPV, which was perpetrated by other staff. Whilst these groups shared some similar findings related to knowledge and confidence, the main thrust of the interventions and measures was concerned with self‐efficacy. Significant improvements were found by Schaefer (2014) for non‐verbal abuse recognition (M = 1.49, SD = .507); t (67) =2.679, *p* = .009 and Sanner‐Stiehr and Ward‐Smith (2015) with self‐efficacy significantly improved (*p* < .002).

##### Knowledge

Knowledge refers to the registered nursing students' knowledge and capacity to recognize instances of WPV or incivility. Tecza et al. (2018) had the most robust findings of knowledge measured as students' capacity to recognize incivility (significant statistical differences were found in the mean scores of 7 of the 12 analysed using t‐tests). Significance was considered when a p‐value of <.05 was found. Schaefer (2014) found a statistically significant increase in knowledge in only one measured item of knowledge in the post‐test finding recognition of non‐verbal abuse. The subtle nonverbal exasperated sigh by the nurse (negative behaviour) in response to the student was found to be statistically significant with the score for the intervention group (M = 1.49), SD = .507; t (67) =2.679, *p* = .009 (two‐tailed). Finally, Murray (2020) found there was a statistically significant improvement in self‐confidence scores in recognizing and addressing uncivil behaviours (*p* < .01).

##### Self‐efficacy

Six studies evaluated self‐efficacy as an outcome (Beech & Leather, 2003; Jeong & Lee, 2020; Murray, 2020; Nau et al., 2009; Sanner‐Stiehr, 2018; Sanner‐Stiehr & Ward‐Smith, 2015), with participants indicating that they could confidently manage future encounters with workplace violence. Murray (2020) found medium‐strength evidence that self‐efficacy improved following the intervention pre‐test (M = 3.03, SD = .35) and post‐test (M = 3.38, SD = 0.30), with an eta squared effect of (r = .55). Sanner‐Stiehr and Ward‐Smith (2015) and Sanner‐Stiehr (2018) also found a significant improvement across all 10 of the instruments used (*p* = .00 and effect size .40) and (*p* = .0020), respectively. Similarly, Nau et al. (2009) found statistically significant (*p* = .033) in dealing with aggressive patients, while Jeong and Lee (c^2020^) reported no significant difference (*p* = .437) in self‐efficacy. Beech and Leather (2003) mentioned that personal safety is identified as attitudes, but no precise data regarding self‐efficacy exists.

##### Other

One study (Tecza et al., 2018) indicated that registered nursing students felt respected (*p* = .000), acknowledged (*p* = .000), had a reduction in anxieties with the clinical setting (*p* = .000), felt that registered nurses went out of their way to assist with a registered nursing student's learning (*p* < .002), knew how to improve care (*p* = .000) and had encouragement shown in asking questions of senior staff (*p* < .003) at 6 months post‐intervention. Tecza et al. (2018) was the only study to measure constructs beyond 3 months from the time of intervention and to use empirical data to display outcomes related to the incidence of WPV. Only the study conducted by Jeong and Lee (2020) focused on post‐event support for RNS following incidences of WPV and found that the experimental group showed more statistically significant effects regarding emotion‐focused coping styles in the follow‐up assessment than the control group.

## 5 DISCUSSION

Each reviewed study used a specific theoretical stance ranging from Cognitive behaviour therapy (Sanner‐Stiehr, 2018), knowledge development (Johnston & Fox, 2020; Stephen et al., 2022; Tecza et al., 2018), self‐efficacy and confidence (Murray, 2020; Nau et al., 2009; Sanner‐Stiehr & Ward‐Smith, 2015) that underpin the interventions and supports interpretations of the effectiveness of improved capacity of RNS to manage WPV in the clinical context. The interventions described prepared and improved the skills of RNS's to meet WPV situations with varying success. The lack of longitudinal empirical evidence of WPV incidences fails to provide clear evidence of effectiveness; rather it displays a belief in the potential of decreasing WPV events through improved knowledge, confidence, communication and skills of RNS. This view could be challenged by the mixed success of the interventions described in this review, and as one study stated, an increase in confidence and perceived self‐efficacy of RNS does not necessarily lead to a reduction in WPV events (Nau et al., 2010). Thus, future longitudinal observational research is necessary to guide future practice. A systematic review conducted by Spelten et al. (2020) and focused on organizational interventions for preventing and minimizing aggression towards healthcare workers by patients found very little evidence that organizational interventions focused on pre‐event or event phases may reduce overall aggression compared to practice as usual. The quality of the studies in this review is also of concern, as nearly half (*n* = 6) had a quality score of less than 50%; this lack of quality is compounded by the mostly medium to high assessment of risk of bias.

A recent paper by Dunseth‐Rosenbaum et al. (2023) has made the critical link that is missing in the reviewed papers between interventions and the reduction of WPV events and has clearly described a multi‐layered approach that included interventions, inclusiveness, monitoring of effectiveness and feedback to staff. Dunseth‐Rosenbaum et al. (2023) identified that the reduction of WPV required not only a multifaceted approach but also a “process‐improvement” methodology that engages with staff across all levels within an organization.

Despite the well‐documented incidence of lateral WPV in nursing (Dafny & Beccaria, 2020; Nikstaitis & Simko, 2014; Roberts, 2015), only five of the 13 included studies viewed WPV towards RNS from a lateral violence organizational perspective (Schaefer, 2014; Sanner‐Stieghr & Ward‐Smith, 2015; Tecza et al., 2018; Sanner‐Stiehr, 2018; Murray, 2020). The interventions described in these five papers, with the exception of Tecza et al. (2018) focused on RNS self‐efficacy and capacity to recognize and respond to lateral violence events, a damage control approach rather than one of elimination of lateral violence. Thus, the problem of WPV experienced by RNS in the clinical setting has been consigned to the educational organizations and registered nursing students themselves to develop skills and strategies for management and prevention, which addresses only a portion of the WPV event cycle and aetiology. The much fewer interventions and evaluative evidence that address lateral violence align with a review of lateral violence, bullying and incivility in nursing by Roberts (2015) nearly a decade earlier. Roberts (2015) reviewed lateral violence over the previous three decades and found that the few interventions developed recognized organizational leadership as vital to the understanding and elimination of lateral violence in the workplace (Roberts, 2015). The lack of evidence in support of leadership variables linked with WPV interventions supports the view that nursing still has a long way to go to progress to an enlightened learning culture, where WPV and, specifically, lateral violence is eliminated.

The review aimed to systematically investigate the effectiveness of interventions for managing or preventing workplace violence experienced by registered nursing students (RNS) and the provided support following incidents of violence during clinical placement. The support received by RNS following a WPV incidence was found only in one paper (Jeong & Lee, 2020) that addressed the issue of post‐event support despite well‐documented evidence of the link between WPV experiences, post‐traumatic stress disorder and negative psychological impact (Wang et al., 2022). This paucity of evidence further highlights the need for further research, development and implementation of multi‐faceted interventions and processes, improvement frameworks that provide nurses, including our most vulnerable RNSs, with appropriate knowledge and skills to de‐escalate violence events and to be supported following WPV events irrespective of the perpetrator of the violence. There is an urgent need to be proactive at both the educational and organizational levels so that the WPV and lateral violence so often embedded within the culture of nursing (Vidal‐Alves et al., 2021) is addressed with a much deeper conceptual framework that moves beyond poorly supported interventions and a singular WPV recipient focus.

## 6 LIMITATIONS

Studies included in this review differed considerably in methods, outcome measures and the instruments used. Therefore, it was not possible to pool data. Several of the studies had single measurements soon after the delivery of the intervention, preventing conclusions from being drawn about the effectiveness and long‐term effects of the interventions. A reliance on the self‐reports of study participants when measuring the outcome effects of interventions such as those in the included studies in this review can increase the risk of bias. Studies with low quality scores were not excluded from this review, and these limitations must be taken into consideration when interpreting results and conclusions. Interventions were, in some cases, evaluated after participants undertook clinical placement. Therefore, responses may have been influenced by the learning that occurred during clinical placement experiences.

### 6.1 Implication for research practice

A direct comparison of the interventions from the included studies is not available to support the efficacy of one intervention over another. However, education providers can adapt and apply aspects of the evidence presented that are best suited to their environment. The development and utilization of standardized instruments to evaluate the outcomes of violence training, in addition to longitudinal studies to measure how knowledge and skills are used during clinical placement, are research priority areas. Higher education providers and the nursing profession should work together in developing resources and strategies for RNS.

Further research is needed to explore strategies for addressing lateral violence, which is often ingrained in workplace culture, and to determine necessary support measures for RNS following an incident of WPV.

## 7 CONCLUSIONS

Registered nursing students frequently experience workplace violence during clinical placement with such dire consequences. This review identified educational interventions with some positive outcomes indicating the level of preparedness for addressing WPV for RNS, which includes knowledge gains, increased confidence, self‐efficacy and the support received by RNS following a WPV incidence (the second aim of this systematic review). However, this review found that no studies used the incidence of violence as a measure of effectiveness (the first aim of this systematic review). The absence of studies evaluating whether learning had resulted in a decrease in workplace violence in the clinical areas is not surprising given the difficulty in controlling real‐life conditions so that desired behaviours can be demonstrated. There is an opportunity for education providers and healthcare organizations to work together to build on these RNS‐focused findings and implement and evaluate educational programs which factor in the situational and contextual variables of the clinical environment and address the multifaceted aspects of workplace violence.

## AUTHOR CONTRIBUTIONS

All authors (HD, NW, CJC, SJ, VP, AA, CP, SB, CM) contributed. HD designed and supervised the study, and SB performed the search strategy. All authors critically reviewed, revised and approved the submission of the review.

## FUNDING INFORMATION

The authors acknowledge that they have not received funding for this systematic review.

## CONFLICT OF INTEREST STATEMENT

The proposed publication does not directly or indirectly concern any commercial product. The authors have no financial or other interests which conflict with this work. Review authors who are authors of any work under consideration for inclusion in this review will not be involved in review processes concerning that study.

### PEER REVIEW

The peer review history for this article is available at https://www.webofscience.com/api/gateway/wos/peer‐review/10.1111/jan.16357.

## IMPACT

Educational institutions and healthcare organizations can customize and combine interventions at both institutional and organizational levels to manage workplace violence in the specific environment encountered by registered nursing students.

## NO PATIENT OR PUBLIC CONTRIBUTION

As this study is a systematic review.

## Data Availability

The data that supports the findings of this study are available in the supplementary material of this article.

## APPENDIX A Search strategy

### A.1

Preliminary search conducted using the database(s): Ovid MEDLINE®, run on 27th of February 2023.

**Table:**

Ovid MEDLINE(R) and Epub ahead of print, in‐process, in‐Data‐Review & Other non‐Indexed Citations, daily and versions <1946 to February 27, 2023>		
Search	Query	Records retrieved
1	Students, Nursing/	29,858
2	Exp Education, Nursing/	88,824
3	((student* or educat* or undergrad* or trainee or pre‐registrat* or “pre registrat*”) adj3 nurs*).tw,kf.	59,861
4	Or/1–3	121,196
5	Violence/or gender‐based violence/or physical abuse/or workplace violence/	37,312
6	Aggression/ or agonistic behaviour/ or bullying/ or cyberbullying/ or exp Racism/ or sexism/ or Sexual Harassment/	54,275
7	(violent or violence or bully* or aggress* or agonistic or agonist or antagonis* or fight* or abuse or harass* or belittle or cyberbully* or abuse or abusive or threat* or brutalis* or incivil* or assault* or sexism or racism or racist).tw,kf.	1,289,516
8	Or/5–7	1,313,069
9	Clinical Clerkship/	5700
10	Preceptorship/	5575
11	(placement* or practicum or rotation* or Preceptorship or clerkship or pre‐place* or “pre place*” or “work place*” or work‐place* or workplace or ward*).tw,kf.	438,999
12	Or/9–11	444,840
13	Clinical study/or exp clinical trial/or controlled clinical trial/or exp randomized controlled trial/or observational study/or comparative study/or multicentre study/	2,942,370
14	Control groups/or cross‐over studies/or double‐blind method/or matched‐pair analysis/or random allocation/or single‐blind method/ or Control Groups/or Matched‐Pair Analysis/or prospective studies/or retrospective studies/	2,010,377
15	Clinical studies as topic/or clinical trials as topic/or case–control studies/or retrospective studies/or cohort studies/or follow‐up studies/or prospective studies/or controlled before‐after studies/or cross‐sectional studies/or interrupted time series analysis/or multicenter studies as topic/	3,161,768
16	(“clinical trial” or “clinical trial, phase i” or “clinical trial, phase ii” or clinical trial, phase iii or clinical trial, phase iv or controlled clinical trial or “multicenter study” or “randomized controlled trial” or clinical study or comparative study or controlled clinical trial or equivalence trial or observational study).pt.	2,941,749
17	(randomized or randomized or randomly or random or placebo or mask* or blind* or trial or placebo or quantitative or cohort or “4 arm” or “four arm” or baseline or consecutive* or “case series” or RCT).tw.	4,127,492
18	((quasi‐experimental or clinical or cluster* or “before and after” or “before‐and‐after” or crossover or “cross‐over” or “crossover” or retrospective or cohort or follow‐up or “follow‐up” or prospective or observation* or pre‐post*) adj5 (trial* or studies or study)).tw.	2,125,779
19	(time* adj2 interrupted).tw.	5480
20	(control* adj4 (Cohort* or group*)).tw.	705,325
21	(mixed‐method* or mixed method*).tw.	37,985
22	Or/13–21	8,440,512
23	4 and 8 and 12 and 22	162
24	Exp animals/not humans.sh.	5,094,662
25	23 not 24	162
